# Evaluating Tuberculosis Case Detection in Eritrea

**DOI:** 10.3201/eid1310.061279

**Published:** 2007-10

**Authors:** Marieke J. van der Werf, Mineab Sebhatu, Martien W. Borgdorff

**Affiliations:** * KNCV Tuberculosis Foundation, The Hague, the Netherlands; † University of Amsterdam, Amsterdam, the Netherlands; ‡ Ministry of Health, Asmara, Eritrea

**Keywords:** Tuberculosis, case detection rate, prevalence survey, evaluation, Eritrea, dispatch

## Abstract

We used results from a national tuberculosis prevalence survey in Eritrea to calculate case detection rate (CDR) and compared it with the published CDR. The CDR obtained from the survey was ≈40%, whereas the CDR published by the World Health Organization was 3× lower (14%).

During the World Health Assembly in 1991, 2 targets were set for tuberculosis (TB) control: to detect 70% of all new sputum smear–positive cases arising each year and to successfully treat 85% of these cases (*1*). For assessment of the first target, case detection rate (CDR) is used; CDR is the number of cases reported divided by the number of incident cases estimated for that year. In Africa in 2004, the range of CDRs for new smear-positive TB patients was 14%–115% in different countries (*2*). The CDR is uncertain for many African countries because information for estimating the incidence is outdated or unavailable. The most recent national TB prevalence surveys were performed from 1955 through 1960; they covered 11 countries and a population of ≈40,000 (*3*). Since then, TB treatment has become widely available, and the emergence of HIV has had a substantial effect on TB incidence (*4**,**5*).

Recently, a TB prevalence survey was performed in Eritrea, a country with a population of 3 million, located in the Horn of Africa (*6*). The survey determined the prevalence of sputum smear–positive TB by examining sputum samples of persons >15 years of age. To assess the performance of Eritrea’s TB program, we calculated the CDR by using information obtained from the survey and compared this CDR to published estimates.

## The Study

The national TB prevalence survey in Eritrea was conducted from February through October 2005 (*6*). In 40 selected villages, a census (which included information about sex and age) was taken of ≈875 persons in each village. All persons >15 years of age were asked to provide a morning and a spot sputum sample. Persons were informed about the survey and could refuse participation. The study protocol for the prevalence survey was approved by the Ministry of Health.

The specimens were examined by fluorescence microscopy. Samples positive by fluorescence microscopy were reexamined by light microscopy for confirmation. Persons who had 2 positive sputum samples were informed about the test results and referred for treatment. Those who had 1 positive sputum sample were referred to a nearby healthcare facility for further smear examination. If results of smear examination were negative, thoracic radiographs were taken and evaluated by 2 experienced radiologists. The case definition for a sputum smear–positive case was at least 2 sputum specimens positive for acid-fast bacilli by Ziehl-Neelsen staining and microscopy or at least 1 sputum specimen positive for acid-fast bacilli and radiographic abnormalities consistent with active pulmonary TB (classification of the National Tuberculosis Control Program in Eritrea).

Using the prevalence estimate obtained from the survey and 2 different models, we calculated the CDR for 2004. In model 1, described by Styblo, CDR = (notification rate/prevalence rate) / (0.5 + 0.83 × [notification rate/prevalence rate]) (*7**,**8*). In model 2, described by Dye et al., CDR = (notification rate/prevalence rate) / ([notification rate/prevalence rate] + 0.5) (*9**,**10*). We then compared the calculated CDR with the CDR estimated by the World Health Organization (WHO) to evaluate whether comparable conclusions about TB case detection would be obtained.

A total of 38,047 persons were included in the prevalence survey. Of those >15 years of age, 18,152 (94.6%) provided at least 1 sputum sample (Figure). The prevalence of new smear-positive TB was estimated at 90/100,000 (95% confidence interval [CI] 35–145/100,000) in persons >15 years of age. In 2005, 44.7% of the Eritrean population was <15 years of age (*11*), which resulted in an overall new smear-positive TB prevalence of 50/100,000 (95% CI 19–80/100,000) under the assumption of no cases in persons <15 years of age.

**Figure:**
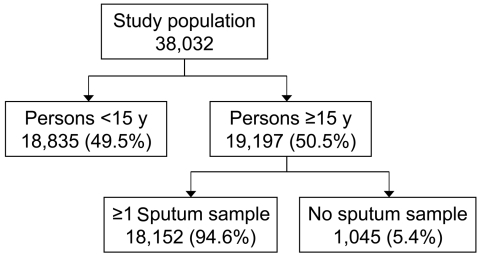
Summary of tuberculosis prevalence survey in Eritrea, 2005.

In 2004, 17/100,000 new smear-positive cases were reported (*2*). The calculated CDR from model 1 was 43% and from model 2 was 40%. The 2004 CDR published by WHO was 14%.

## Conclusions

For Eritrea, the CDR provided by WHO is considerably lower than that calculated from the results of the national TB prevalence survey. Both estimates indicate that Eritrea has not reached the 70% target for case detection. However, the WHO estimate suggests that the program needs to improve case detection by a factor of 5, whereas the survey estimate suggests that case detection needs to be improved by a factor of 1.6. Two explanations may account for the large difference: 1) the CDR derived from the TB prevalence survey is too high because of an underestimation of the prevalence of smear-positive TB, or 2) the CDR estimate published by WHO is too low because of an overestimation of the incidence of smear-positive TB.

In the national TB prevalence survey, measures were taken to ensure high quality of the results; e.g., training of data collectors, repeat census taking, reexamination of all slides found positive on fluorescence microscopy, and reexamination of a 5% random sample of the negative slides. Persons who had smear-positive TB may have been missed because they did not provide a specimen; however, because only 5% of eligible persons did not provide a specimen, this can explain only a slight underestimation. Furthermore, recorded reasons for not providing a specimen seem to be unrelated to a higher chance of having TB. The quality of the provided specimens may have been suboptimal because instructing and motivating persons to provide a sputum sample is challenging. For diagnosis of TB, microscopic examination of saliva is less sensitive than examination of sputum; however, in ≈50% of saliva samples from patients with a positive sputum sample, bacilli can be demonstrated (*12**,**13*). For 27,647 samples that appeared to be saliva, smear–positive results were obtained for 12. Assuming that only 50% were detected, a maximum of 12 smear-positive TB patients may have been undetected. Taking this into account results in a prevalence of 87/100,000. Using this estimate, model 1 provides a CDR of 30% and model 2 a CDR of 28%; both figures are still substantially higher than the WHO CDR of 14%. The possibility that persons who provided a saliva sample were not able to produce a sputum sample because they did not have pathologic pulmonary changes should also be taken into consideration. If so, the estimated prevalence is correct.

Estimation of the incidence of smear-positive TB in Eritrea is complicated by the fact that no data from tuberculin or prevalence surveys were available. The only data available for Eritrea were reporting data, which experts assessed as being of low quality (*14*). Use of this limited information will result in an uncertain incidence estimate, which may result in an unreliable CDR.

For most countries in Africa, little information is available for estimating the prevalence of disease and progress towards the Millennium Development Goals (http://unstats.un.org/unsd/mi/mi_goals.asp, accessed 2006 Aug 30). On the basis of case reporting, TB was rightly declared an emergency by African health ministers at the WHO Africa Regional Committee in Maputo in 2005 (*15*)*.* To be able to fight this emergency, more reliable information about the prevalence of TB in Africa is needed. Furthermore, for global TB control, reliable information about the TB epidemic in Africa is needed because 28% of the incident smear-positive cases occurred in the WHO African region in 2004 (*2*).

In conclusion, the example of Eritrea shows that a large gap may exist between available estimates of TB prevalence and actual TB prevalence in Africa. National TB prevalence surveys in Africa would help provide better information on TB prevalence and case detection.
